# The neuroprotective properties of GLP‐1R agonists correlate with their ability to cross the blood‐brain barrier

**DOI:** 10.1002/alz.088069

**Published:** 2025-01-09

**Authors:** Christian Holscher

**Affiliations:** ^1^ Henan Academy of Innovations in Medical Science, Zhengzhou, Henan China

## Abstract

**Background:**

Glucagon‐like peptide 1 (GLP‐1) is a peptide hormone that plays several physiological roles in treating diabetes and in protecting the brain. Recent clinical trials testing 4 different GLP‐1 class drugs in phase 2 trials showed a clear correlation between neuroprotection and the ability to cross the BBB. Exenatide and Lixisenatide both showed excellent protective effects in patients Parkinson’s disease (PD) and both drugs can readily cross the BBB. Liraglutide showed limited effects and can only cross the BBB at a low rate. A pegylated form of exenatide NLY01 did not show any effects in a clinical trial and the drug does not cross the BBB.

**Method:**

We tested a novel dual GLP‐1/GIP agonist that can cross the BBB easily and compared it to Semaglutide, a GLP‐1 class drug on the market to treat diabetes in the 3xFAD tg mouse model of AD. Water maze tests and in vivo electrophysiology had been conducted, and synapses quantified using the GOLGI method. Biomarkers were analyzed by histology and western blot to test for inflammation.

**Result:**

In a direct comparison, the dual agonist was superior to Semaglutide in improving learning and memory, in vivo LTP in the hippocampus, activation of microglia and release of pro‐inflammatory cytokines. Synapse numbers were enhanced by both drugs.

**Conclusion:**

The results demonstrate that enhanced BBB penetration of GLP‐1 class drugs shows superior neuroprotective effects in a 3xtg mouse model of AD. This mirrors the observations from clinical trials in PD patients that show enhanced protection with drugs that can cross the BBB better.

